# Feasibility of cardiac output measurements in critically ill patients by medical students

**DOI:** 10.1186/s13089-020-0152-5

**Published:** 2020-01-08

**Authors:** Geert Koster, Thomas Kaufmann, Bart Hiemstra, Renske Wiersema, Madelon E. Vos, Devon Dijkhuizen, Adrian Wong, Thomas W. L. Scheeren, Yoran M. Hummel, Frederik Keus, Iwan C. C. van der Horst

**Affiliations:** 1 Department of Critical Care, University of Groningen, University Medical Center Groningen, P.O. Box 30.001, 9700 RB Groningen, The Netherlands; 2 Department of Anaesthesiology, University of Groningen, University Medical Center Groningen, Groningen, The Netherlands; 3 Department of Anaesthesia and Intensive Care, Royal Surrey Hospital, Guildford, UK; 4 Department of Cardiology, University of Groningen, University Medical Center Groningen, Groningen, The Netherlands; 5 Department of Intensive Care, Maastricht University Medical Center+, University Maastricht, Maastricht, The Netherlands

**Keywords:** Ultrasonography, Medical students, Critical care, Intensive care unit, Cardiac output

## Abstract

**Background:**

Critical care ultrasonography (CCUS) is increasingly applied also in the intensive care unit (ICU) and performed by non-experts, including even medical students. There is limited data on the training efforts necessary for novices to attain images of sufficient quality. There is no data on medical students performing CCUS for the measurement of cardiac output (CO), a hemodynamic variable of importance for daily critical care.

**Objective:**

The aim of this study was to explore the agreement of cardiac output measurements as well as the quality of images obtained by medical students in critically ill patients compared to the measurements obtained by experts in these images.

**Methods:**

In a prospective observational cohort study, all acutely admitted adults with an expected ICU stay over 24 h were included. CCUS was performed by students within 24 h of admission. CCUS included the images required to measure the CO, i.e., the left ventricular outflow tract (LVOT) diameter and the velocity time integral (VTI) in the LVOT. Echocardiography experts were involved in the evaluation of the quality of images obtained and the quality of the CO measurements.

**Results:**

There was an opportunity for a CCUS attempt in 1155 of the 1212 eligible patients (95%) and in 1075 of the 1212 patients (89%) CCUS examination was performed by medical students. In 871 out of 1075 patients (81%) medical students measured CO. Experts measured CO in 783 patients (73%). In 760 patients (71%) CO was measured by both which allowed for comparison; bias of CO was 0.0 L min^−1^ with limits of agreement of − 2.6 L min^−1^ to 2.7 L min^−1^. The percentage error was 50%, reflecting poor agreement of the CO measurement by students compared with the experts CO measurement.

**Conclusions:**

Medical students seem capable of obtaining sufficient quality CCUS images for CO measurement in the majority of critically ill patients. Measurements of CO by medical students, however, had poor agreement with expert measurements. Experts remain indispensable for reliable CO measurements.

*Trial registration* Clinicaltrials.gov; http://www.clinicaltrials.gov; registration number NCT02912624

## Background

Critical care ultrasonography (CCUS) is a deliberately focused examination, aimed at rapidly answering straightforward clinical questions [1]. In the field of emergency and critical care medicine, CCUS is increasingly used to guide interventions in critically ill patients in various settings by experts and novices [2–14]. The training process required for users to attain competency in CCUS has varied widely between studies, reflecting the diversity in CCUS training between centers. Similarly, there is variability among statements from stakeholders regarding the type of training, the required number of hours spent and examinations performed by the trainee to achieve competency in CCUS [15–17]. However, besides these disparities, individual physicians struggle with barriers to its use, such as perceived difficulty in obtaining adequate technical skills [13], limitations in training, need (perceived and real), and costs [6, 14].

One valuable CCUS hemodynamic measurement is the determination of the cardiac output (CO), especially if the patient is in circulatory shock [18]. Circulatory shock occurs in one-third of patients admitted to the ICU [19], so being able to perform CCUS and measure CO is of importance. However, CO measurement by CCUS is considered an advanced level CCUS skill [20, 21]. Whether trained novices (e.g., medical students or other less experienced physicians) are able to obtain reliable CO measurements has not yet been investigated. In a convenience sample of 100 adult patients in the emergency department (ED), two ultrasound-naive ED physicians were able to measure CO by ultrasonography accurately [22]. Another study in the ED with a convenience sample of 80 patients, however, showed poor agreement in CO measurement by an emergency ultrasound fellow compared to an emergency cardiology fellow [23]. At the start of our study there were no data on medical students performing CO measurements by CCUS in critically ill patients, although medical students have been shown to be capable of performing CCUS after limited training [24]. To our knowledge, only one small study investigated CCUS by medical students on a (cardiac) intensive care unit, and CO was not measured (see Additional file 1) [3].

The aim of this study was to explore the feasibility of a limited CCUS examination, consisting of CO measurements, performed by medical students in a protocolized manner, in critically ill patients. In addition, the quality of images required to calculate CO and the accuracy of CO measurements compared to those obtained by echocardiography experts were analyzed.

## Methods

The Simple Intensive Care Studies (SICS)-I was a prospective, observational cohort study which followed a published protocol and statistical analysis plan (Clinicaltrials.gov; NCT02912624). The SICS-I was developed to unravel the diagnostic and prognostic value of a comprehensive selection of clinical, hemodynamic, and biochemical variables in critically ill patients, and details have been described elsewhere [25, 26]. All acutely admitted adults with an expected ICU stay over 24 h were included. Patients were excluded when admission was planned and if clinical care interfered with acquiring research data (e.g., mechanical circulatory support). The local institutional review board approved the study (M15.168207).

### Data collection and training

All patients underwent CCUS within 24 h of ICU admission. Detailed information on the CCUS performed can be found in Additional file 1. Patients were enrolled by 4th-year to 6th-year medical students of a 6-year medical school program. The training consisted of self-study on theoretical fundamentals and two practical sessions of at least 2 h in total to learn how to operate the General Electric Vivid-S6 mobile ultrasonography machine using the cardiac phased-array probe (see Appendix in Additional file 1 for detailed information). The theoretical self-study on how to perform CCUS and measure the CO consisted of study of the protocol (Additional file 1), a website on the principles of echocardiography [27], and international guidelines [28, 29]. This information became available 2 weeks before participation of the medical students. During the practical sessions, medical students learned to obtain the parasternal long axis (PLAX), apical four-chamber (AP4CH), and apical five-chamber (AP5CH) views, among others. The medical students alternated with obtaining the views and measurements of CO during the practical sessions. All medical students received at least 2 h hands-on training from cardiologist-intensivists (GK and IVDH).

Views and images were obtained randomly during the respiratory cycle and/or phase of mechanical ventilation. In case of any arrhythmias, the average of multiple measurements over five heartbeats was taken.

The first 20 CCUS images and measurements of each medical student were supervised by medical students who had independently performed more than 50 CCUS examinations. After 20 scans, CCUS medical students were allowed to conduct/perform CCUS unsupervised, since previous studies showed acceptable capability for acquiring images beyond 20 exams [30].

### Validation and definitions

For quality control, echocardiography technicians from an independent core laboratory (Groningen Image Core Lab, UMCG, Groningen, the Netherlands, http://www.g-icl.com) assessed all CCUS images and measurements obtained by the medical students according to the study protocol. If the images were obtained according to guideline standards, the LVOTd and VTI were independently remeasured and CO recalculated [28, 29]. Core laboratory technicians, who we refer to as *experts* throughout this report, were blinded to all other clinical measurements. The experts did not perform any CCUS examination.

### Outcomes, index test and reference standard

The number of patients where CCUS could not be performed and reasons for unobtainable images by the medical students were reported. Patients were excluded from the analysis if, for research purposes, experts would also not be able to perform CCUS (i.e., drains, subcutaneous emphysema, surgical dressing/wounds). The number of patients in which CCUS images of PLAX or AP5CH were obtained was analyzed [28, 29]. Proportion of patients was reported wherein the CCUS images assessed by the experts was of insufficient quality for CO measurement.

We also evaluated the accuracy of CO measurements by medical students (CO_medical student_) compared to CO measurements by experts (CO_expert_). Moreover, the two components needed for CO calculation (i.e., LVOTd or VTI) were assessed to determine possible differences between medical students’ and experts’ measurements.

Sensitivity analyses were done with baseline characteristics to investigate reasons why experts could not measure a CO.

### Sample size and missing data

Due to the observational nature of this study, no formal power calculation was performed. For the accuracy analysis on CO measurements, we only included patients if CO was measured by both medical students and experts.

### Statistical analysis

Data were presented as mean with standard deviation (SD) when normally distributed or as median with interquartile ranges (IQR) in case of skewed data. Dichotomous and categorical data were presented in proportions. Intraclass correlation coefficients (ICC) were calculated to assess the concordance between the measurements made by the medical students and the experts. Bland–Altman analysis was performed to assess agreement of medical student versus expert measurements by calculating mean and SD of the differences, the 95% limits of agreement (LOA) (= mean of the difference ± 1.96 × SD of the difference), and the percentage error [31]. In method comparison studies, a percentage error of 30% is considered acceptable if the error of the test and the reference method is 20%, which is the case when using the thermodilution method to calculate CO [32]. Since there is no reference for CCUS, and only one method was used with comparison between the observers, a percentage error of less than 20% was defined as clinically acceptable. This would mean that the CO difference between medical students and experts would be less than 0.5 L min^−1^ in the lower end of the CO spectrum (e.g., when the experts measured a CO of 2.5 L min^−1^, a CO of 2.0–3.0 L min^−1^ by the medical student would be clinically acceptable). An alpha error of 0.05 was used to indicate statistical significance. Statistical analyses were conducted using STATA version 15.0 (StataCorp, College Station, USA).

## Results

### CCUS acquisition and images

**Fig. 1:**
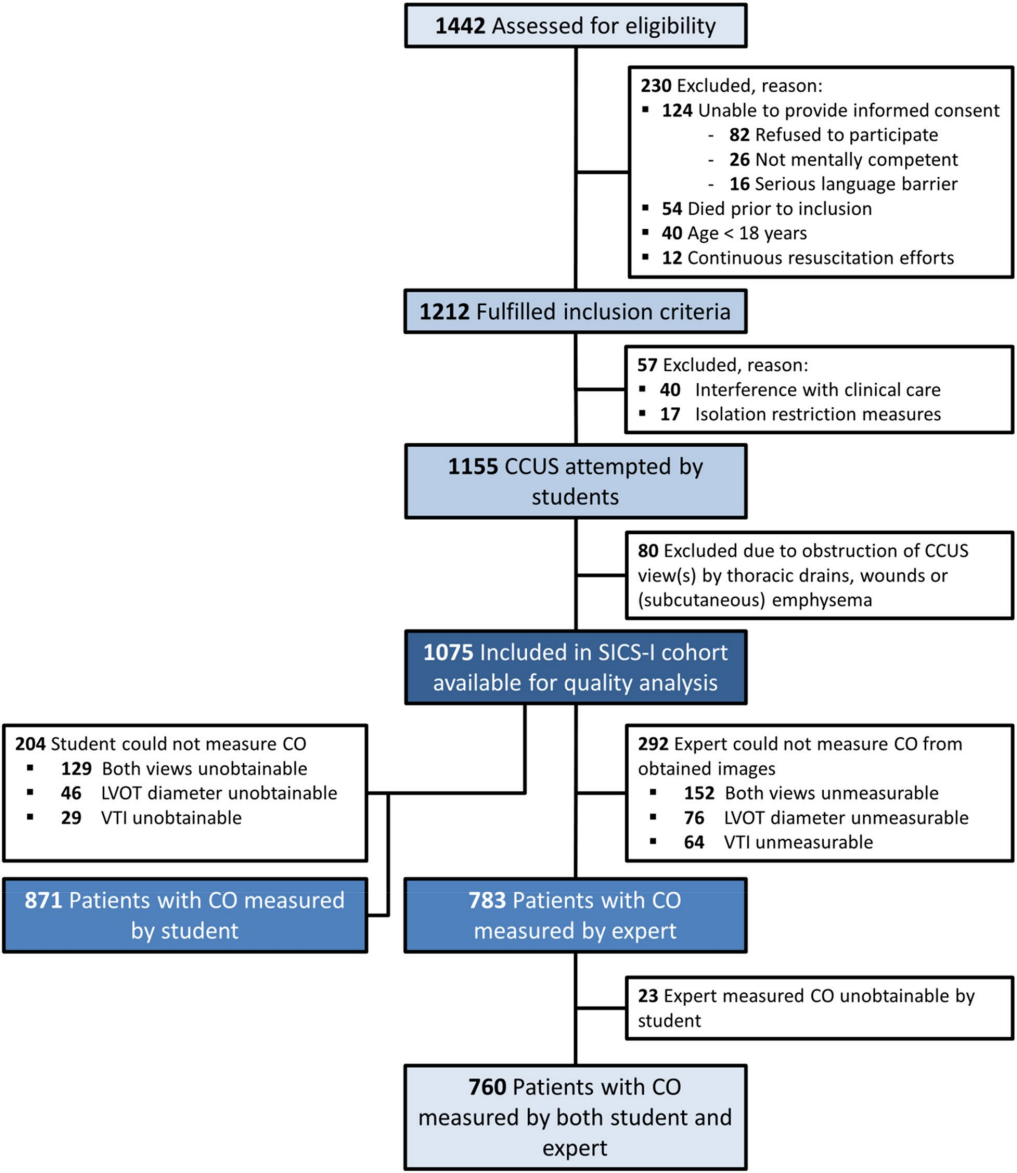
Flow diagram of the Simple Intensive Care Studies-I (SICS-I). *ICU* intensive care unit, *CCUS* critical care ultrasonography, *CO* cardiac output, *LVOT* left ventricular outflow tract, *VTI* velocity time interval

Between March 27th, 2015 and July 22nd, 2017, 16 medical students were involved in the study and 1212 patients fulfilled inclusion criteria. Of these, in a total of 1155 patients CCUS was performed, as in 40 patients there was interference with clinical care during the first 24 h of admission (e.g., the patient was in severe hemodynamic instability or an intervention was being performed) and 17 patients had isolation restriction measures. Of these 1155 patients, in 80 patients, clinical conditions (i.e., thoracic drains, wounds, or subcutaneous emphysema) prohibited the image acquisition by CCUS, leaving 1075 patients with ultrasonography data (Fig. 1).

The medical students deemed both LVOTd and VTI unmeasurable (i.e., images were of too low a quality and no or few structures could be identified) in 129 patients (12%), the LVOTd in 46 patients (4.2%), and the VTI in 29 patients (2.6%). The parasternal short axis view did not provide any additional measurements when the LVOTd was unmeasurable in the PLAX view. Thus, 204 patients (19%) out of 1075 had no CO measurement, leaving a total of 871 patients (81%) with a measured CO by medical students.

### CCUS quality of images

The experts used the images obtained by the medical students and were unable to measure both the LVOTd and VTI in 152/1075 (14%), LVOTd in 76/1075 (7.1%), and VTI in 64/1075 (6.0%). While the experts deemed more measurements to be impossible in the obtained images compared to the medical students, the experts were also able to add 23 CO measurements in patients where medical students judged the images to be of too poor a quality and consequently did not perform the measurements. In total, the experts measured CO in 783 patients (73%). Comparisons of CO measurements by medical students and experts were possible in 760 (71%) out of 1075 patients in case of adequate image quality (Fig. 1).

Differences in patient baseline characteristics were found between the group in which experts could measure a CO and the group in which experts could not measure a CO (see Table 1). Patients without CO measured by experts were characterized by older age, greater illness severity (reflected in higher APACHE IV scores), higher heart rate, greater prevalence of chronic obstructive pulmonary disease (COPD), higher rates of mechanical ventilation, greater likelihood of being post-operative, and higher vasopressor dose.

**Table 1:** Patient characteristics separated on the presence or absence of an expert-measured cardiac output (*n* = 1075)

	Patients without CO measurement (*n* = 292)	Patients with CO measurement (*n* = 783)	*p*-values
Age (years)	64 ± 13	61 ± 15	0.004
Male gender	190 (65%)	484 (62%)	0.33
BMI (kg m^−2^)	26.9 ± 5.3	26.9 ± 5.6	0.96
Respiratory rate (bpm)	18 ± 6	18 ± 6	0.88
Mechanical ventilation	194 (66%)	438 (56%)	0.002
PEEP (cm H_2_O)	7 (5, 8)	7 (5, 8)	0.83
SBP (mmHg)	113 ± 25	120 ± 25	< 0.001
DBP (mmHg)	59 ± 12	60 ± 12	0.44
MAP (mmHg)	76 ± 14	79 ± 14	0.014
Heart rate (bpm)	91 ± 22	87 ± 21	0.002
Atrial fibrillation	22 (8%)	56 (7%)	0.91
Norepinephrine	168 (58%)	361 (46%)	< 0.001
CVP (mmHg)	9 (4–12)	9 (5–13)	0.84
Lactate (mmol L^−1^)	1.5 (1.0–2.5)	1.3 (0.9–2.1)	< 0.001
Consciousness			
Alert	75 (26%)	254 (32%)	0.018
Reacting to voice	49 (17%)	154 (20%)	
Reacting to pain	22 (8%)	67 (9%)	
Unresponsive	146 (49%)	308 (39%)	
COPD	54 (18%)	88 (11%)	0.002
Acute surgery	108 (37%)	230 (29%)^a^	0.017
Post-cardiothoracic surgery	40 (14%)	48 (6%)	< 0.001
SAPS-II	49 ± 17	46 ± 17	0.004
APACHE IV score	80 ± 30	75 ± 29	0.017
90-day mortality	80 (27%)	217 (28%)	0.97

*APACHE* acute physiology and chronic health evaluation, *BMI* body mass index, *bpm* beats per minute, *CO* cardiac output, *CVP* central venous pressure, *DBP* diastolic blood pressure, *MAP* mean arterial pressure, *PEEP* positive end-expiratory pressure, *SAPS* simple acute physiology score, *SBP* systolic blood pressure

^a^Significant overlap with cardiothoracic surgery

### Comparison of CO measurement by medical students and experts

**Fig. 2:**
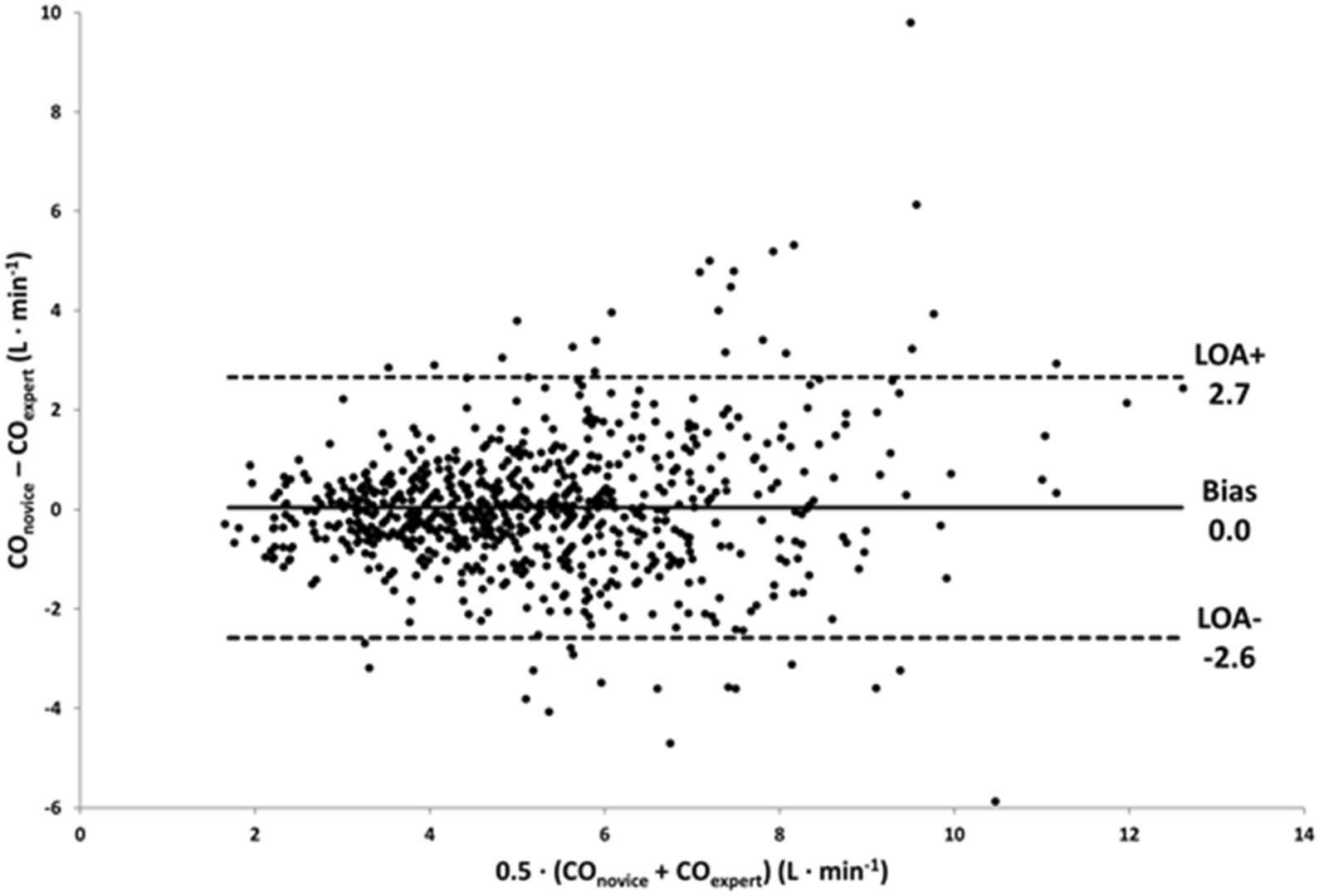
Bland–Altman plot showing the comparison between cardiac output measured by medical students (CO_medical student_) and core lab experts (CO_expert_). The mean bias between CO_expert_ and CO_medical student_ and the upper and lower limits of agreement (LOA) are presented. The figure clearly shows the widening of the LOA in both directions with increasing CO

The mean CO_medical student_ was 5.2 ± 2.0 L min^−1^ and CO_expert_ was 5.2 ± 1.8 L min^−1^ (*p* = 0.44). Bland–Altman analysis demonstrated a bias of − 0.0 L min^−1^ (95% CI − 0.06 to 0.13) with limits of agreement of − 2.6 L min^−1^ (95% CI − 2.7 to − 2.4) to 2.7 L min^−1^ (95% CI 2.5–2.8) (Fig. 2). Plotting a regression line in the Bland–Altman plot showed a proportional bias of 2%. The percentage error was 50% (95% CI 47–53). The ICC was 0.75 (95% CI 0.72–0.78).

### Comparison of LVOTd and VTI measurements by medical students and experts

The medical students measured 900 LVOTd and the experts 847. There were 815 paired LVOTd measurements. Mean LVOTd by medical students (LVOTd_medical student_) was 2.06 ± 0.24, whereas the mean of the LVOTd measured by experts (LVOTd_expert_) was 2.09 ± 0.18 (*p* < 0.001). Bland–Altman analysis showed a bias of 0.0 cm (95% CI 0.0–0.0) with limits of agreement of − 0.5 cm (95% CI − 0.5 to − 0.4) to 0.4 cm (95% CI 0.4–0.4) (see Additional file 1). The percentage error was 21% (95% CI 20–23). There was a proportional bias of 20% (0.41 cm). The ICC was 0.43 (95% CI 0.37–0.48).

The medical students measured 917 VTI and the experts 859. There were 840 paired VTI measurements. Mean VTI by medical students (VTI_medical student_) was 19.0 ± 5.6 cm compared to 18.5 ± 5.4 cm of the experts (VTI_expert_) (*p* < 0.001). Bland–Altman analysis showed a bias of 0.5 cm (95% CI 0.4–0.7) with limits of agreement of − 5.0 cm (95% CI − 5.3 to − 4.6) to 6.1 cm (95% CI 5.7–6.4) (see Additional file 1). The percentage error was 30% (95% CI 28–31). The ICC was 0.86 (95% CI 0.84–0.88).

## Discussion

In this large prospective ICU cohort study with CCUS, we found that, after dedicated training, medical students were able to acquire a CO measurement in three out of every four patients (871 of 1155 patients). This finding is of interest considering that the medical students were ultrasound naïve, the CO measurement is considered an advanced CCUS skill, and the ICU population is known for technical difficulties in acquiring ultrasound images. In a minority of ICU patients (80 of the 1155 patients) CCUS was not possible due to clinical conditions hampering image acquisition, leaving 1075 patients with ultrasonography data. The CCUS images obtained by medical students were assessed by experts and rated to be of adequate quality in 73%. Patients (292 of 1075 patients) in which no adequate image quality could be obtained were more often mechanically ventilated, admitted after cardiothoracic surgery or were more severely ill.

Although the students reached a reasonable percentage on image acquisition/quality, our data do not support CO measurements by medical students (after limited training), as comparison to CO measurements by experts showed poor agreement. CCUS concerns more than acquiring the required images and any operator should be aware of the potential errors that can be made with ultrasonography, especially in complex critically ill patients [33]. It is important to note that education on ultrasonography should focus on specific training and quality control on all aspects of ultrasonography in order to achieve accurate measurements [17]. Our results are in line with recommendations by the European Association of Cardiovascular Imaging (EACVI) on point-of-care, problem-oriented focus cardiac ultrasound examination (FoCUS), which state that supervision and quality control by experts are essential for proper and complete examination. Quality control in our study was performed by an accredited echocardiographic laboratory as is recommended in this viewpoint [15].

To be able to compare our results to those of other studies, it is of utmost importance that every step, from eligible patients to the number of patients in which a reliable CO measurement by CCUS is obtained, is presented. Currently these numbers are often lacking, and this leads to varying success rates on the feasibility of CCUS. If reported, results may vary based on differences in ultrasonography training and experience, which impedes a comparison of image acquisition and quality. We found four studies, on measuring CO in critically ill patients by non-experts to compare with our study (see Additional file 1) [22, 23, 34, 35]. In two out of the four studies the operators had previous experience with ultrasonography, but training varied [23, 34]. The setting, sampling, and exclusion criteria may explain the reported high success rate in one study over another [22]. Whether images obtained are of sufficient quality should preferably be judged by independent experts, as two out of four studies did [22, 34]. In one study independent investigators assessed the quality, however, it is not clear if these were experts or not [35]. The percentage of adequate/good-quality images in our study was comparable with Dinh et al. In the study of Betcher et al. and Villavicencio et al., image quality was generally (judged) overall lower. Duration of training or differences in baseline characteristics might explain part of these differences.

The final step to obtain a reliable CO measurement is to measure LVOTd and VTI on images of sufficient quality. Dinh et al. and Lee et al. reported data on measurement quality, and, furthermore, Dinh et al. reported a low bias between sonographers and independent experts. These studies and ours showed lack in precision for CO measurement by novices. Villavicencio et al. compared ultrasonography-derived CO with the transpulmonary thermodilution technique and concluded that there was an acceptable level of agreement between the techniques. Furthermore, they found a high inter- and intra-observer reliability.

Ultrasonography in the acute setting remains challenging, and data regarding novice-based CCUS are limited (see Additional file 1). In our study we chose for medical students as novices (i.e., non-experts), since non-experts constitute the majority of ultrasound trained personnel in an IC and as students would not interfere with daily ICU care. Five studies reported on medical students performing CCUS in critically ill patients (3 in ED setting, 1 in operating theater and 1 in ICU) [3, 7–10]. Four out of the five studies showed that images could be acquired in a promising 82–98% of cases. The studies reporting on image quality showed percentages of (at least) adequate imaging ranging from 89 to 98%, unfortunately by non-independent judging [3, 7]. Furthermore, after training, medical students can adequately interpret images with a very simplified or binary assessment [36]. A number of previous studies employed training curricula for medical students on ultrasonography protocols [37–39]. Four other studies used a point-of-care ultrasonography training program to determine diagnostic performance in various clinical scenarios [36, 40–42]. All studies showed feasibility to train medical students to perform ultrasonography after a relatively short amount of training, which is comparable to the training medical students received in our study.

In previous manuscripts on SICS study data we reported a higher percentage of images judged to be of sufficient quality [25, 26]. The current results showed the percentage of measurements of CO considered of sufficient quality by a core-laboratory and not images with a LVOT and VTI. The high(er) level of quality considered necessary is according to internal protocol and is independently monitored.

### Limitations

First, the proportion of patients with an acoustic window was based on the results of CCUS by medical students only. We did not check if more experienced sonographers were able to retrieve images in these cases, because the design of our study was to obtain images outside patient care. We believe image quality can only be assessed if the observers are blinded for all other study data and are not involved in the patient’s clinical care. Ideally, independent experts perform ultrasonography themselves and make a direct comparison with the medical student. The availability of time and staff outside clinical care in our center was limited, leading us to include all consecutive patients and allow trained medical students to run the study.

Second, we did not check for interindividual variation of skills and quality of CCUS in each medical student who participated in the study, mainly to limit the time of investigation at the bedside.

Third, CCUS of the heart was limited to 2D imaging of the LVOTd, the AP5CH and pulse wave Doppler imaging of the LVOT. Therefore, valvular disease could have been missed.

## Conclusions

Medical students as novices were capable of performing CCUS with adequate image acquisition in the majority of an ICU population of acutely admitted critically ill patients. However, they cannot accurately measure a CCUS-derived cardiac output after limited training. Cardiac output measurements with CCUS in research and daily care should be interpreted with caution if not validated by experts; this is in concordance with the viewpoint of the EACVI on CCUS.

## Supplementary information

**Additional file 1: Figure S1.** Scatter plot of cardiac output measurements of medical students (CO_medical student_) versus core lab (CO_expert_). **Figure S2.** Bland–Altman plot showing the comparison between cardiac output measured by medical students (CO_medical student_) and core lab (CO_expert_). **Figure S3.** Scatter plot of left ventricular outflow tract diameter measurements of medical students (LVOT_medical students_) versus core lab (LVOT_expert_). **Figure S4.** Bland–Altman plot showing the comparison between left ventricular outflow tract diameter measured by medical students (LVOT_medical students_) and core lab (LVOT_expert_). **Figure S5.** Scatter plot of velocity time interval measurements of medical students (VTI_medical students_) versus core lab (VTI_expert_). **Figure S6.** Bland–Altman plot showing the comparison between VTI measured by medical students (VTI_medical students_) and core lab (VTI_expert_). **Table S1.** Overview of existing recent literature on medical students-based ultrasonography in critically ill patients. **Table S2.** Overview of existing recent literature on CO derived ultrasonography in critically ill patients compared to our study. Additional – appendix CCUS protocol.

## Data Availability

The datasets used and/or analyzed during the current study are available from the corresponding author on reasonable request

## Abbreviations

AP5CH: apical five-chamber view
CCUS: critical care ultrasonography
CO: cardiac output
EACVI: European Association of Cardiovascular Imaging
ICU: intensive care unit
IQR: interquartile ranges
LOA: limits of agreement
LVOT(d): left ventricular outflow tract (diameter)
PLAX: parasternal long axis
SD: standard deviation
SICS: Simple Intensive Care Studies
VTI: velocity time integral
