# Supplementary material for: Feasibility of cardiac output measurements in critically ill patients by medical students
Source: Ultrasound J. 2020 Jan 8;12:1. doi: 10.1186/s13089-020-0152-5 (PMC6946766; doi:10.1186/s13089-020-0152-5)
Supplement: Supplementary file 1 — Additional file 1: Figure S1. Scatter plot of cardiac output measurements of medical students (COmedical student) versus core lab (COexpert). Figure S2. Bland–Altman plot showing the comparison between cardiac output measured by medical students (COmedical student) and core lab (COexpert). Figure S3. Scatter plot of left ventricular outflow tract diameter measurements of medical students (LVOTmedical students) versus core lab (LVOTexpert). Figure S4. Bland–Altman plot showing the comparison between left ventricular outflow tract diameter measured by medical students (LVOTmedical students) and core lab (LVOTexpert). Figure S5. Scatter plot of velocity time interval measurements of medical students (VTImedical students) versus core lab (VTIexpert). Figure S6. Bland–Altman plot showing the comparison between VTI measured by medical students (VTImedical students) and core lab (VTIexpert). Table S1. Overview of existing recent literature on medical students-based ultrasonography in critically ill patients. Table S2. Overview of existing recent literature on CO derived ultrasonography in critically ill patients compared to our study. Additional – appendix CCUS protocol. [file 13089_2020_152_MOESM1_ESM.docx]

# Additional ‘Feasibility of cardiac output measurements in critically ill patients by medical students’

**Figure S1. Scatter plot of cardiac output measurements of medical students (CO_medical student_) versus core lab (CO_expert_)**

**Figure S2. Bland-Altman plot showing the comparison between cardiac output measured by medical students (CO_medical student_) and core lab (CO_expert_).**

**Figure S3. Scatter plot of left ventricular outflow tract diameter measurements of medical students (LVOT_medical students_) versus core lab (LVOT_expert_)**

**Figure S4. Bland-Altman plot showing the comparison between left ventricular outflow tract diameter measured by medical students (LVOT_medical students_) and core lab (LVOT_expert_).**

**Figure S5. Scatter plot of velocity time interval measurements of medical students (VTI_medical students_) versus core lab (VTI_expert_)**

**Figure S6. Bland-Altman plot showing the comparison between VTI measured by medical students (VTI_medical students_) and core lab (VTI_expert_).**

**Table S1. Overview of existing recent literature on medical students-based ultrasonography in critically ill patients**

**Table S2. Overview of existing recent literature on CO derived ultrasonography in critically ill patients compared to our study**

**Additional – appendix CCUS protocol**

**Figure S1. Scatter plot of cardiac output measurements of medical students (CO_medical student_) versus core lab (CO_expert_)**


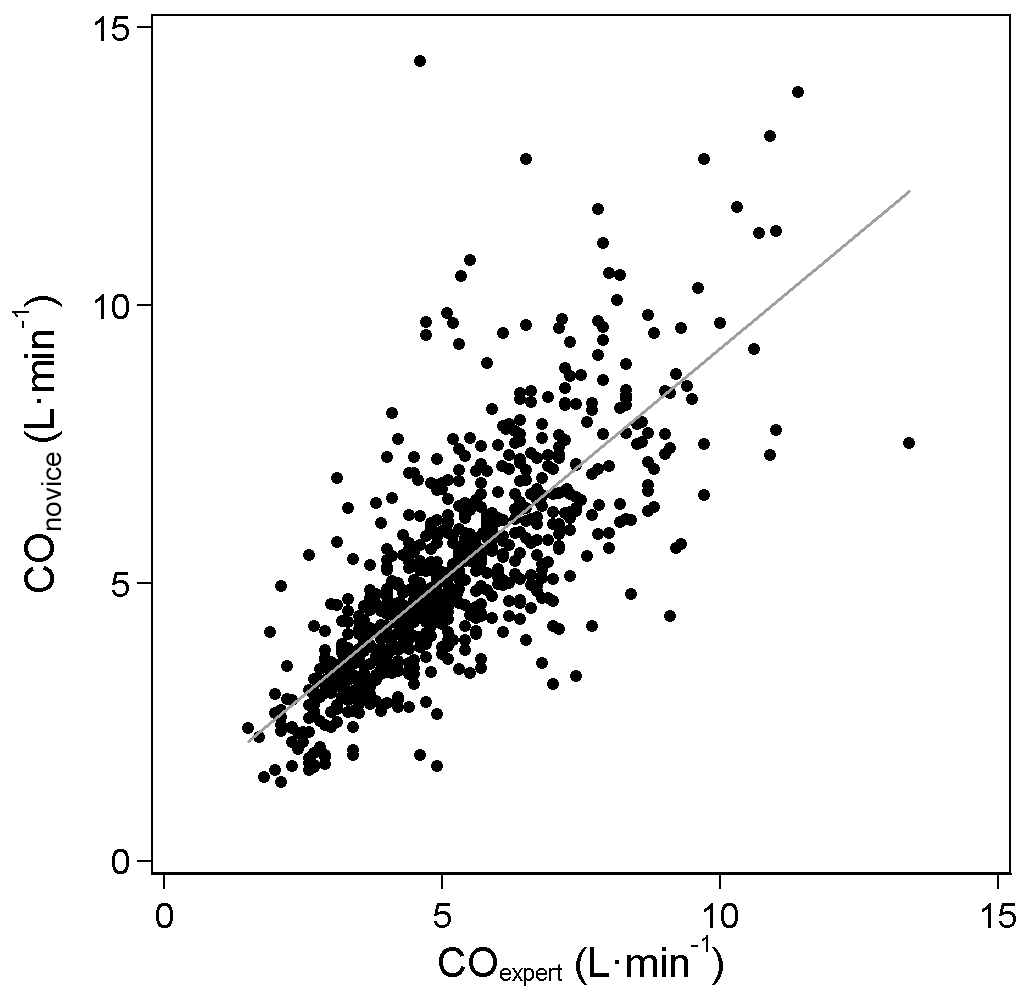


**Figure S2. Bland-Altman plot showing the comparison between cardiac output measured by medical students (CO_medical student_) and core lab (CO_expert_).**

**Legenda**. The mean bias between CO_expert_ and CO_medical student_ and the upper and lower limits of agreement (LOA) are presented. Data presented as difference between the methods plotted as a percentage of their average

**Figure S3. Scatter plot of left ventricular outflow tract diameter measurements of medical students (LVOT_medical students_) versus core lab (LVOT_expert_)**


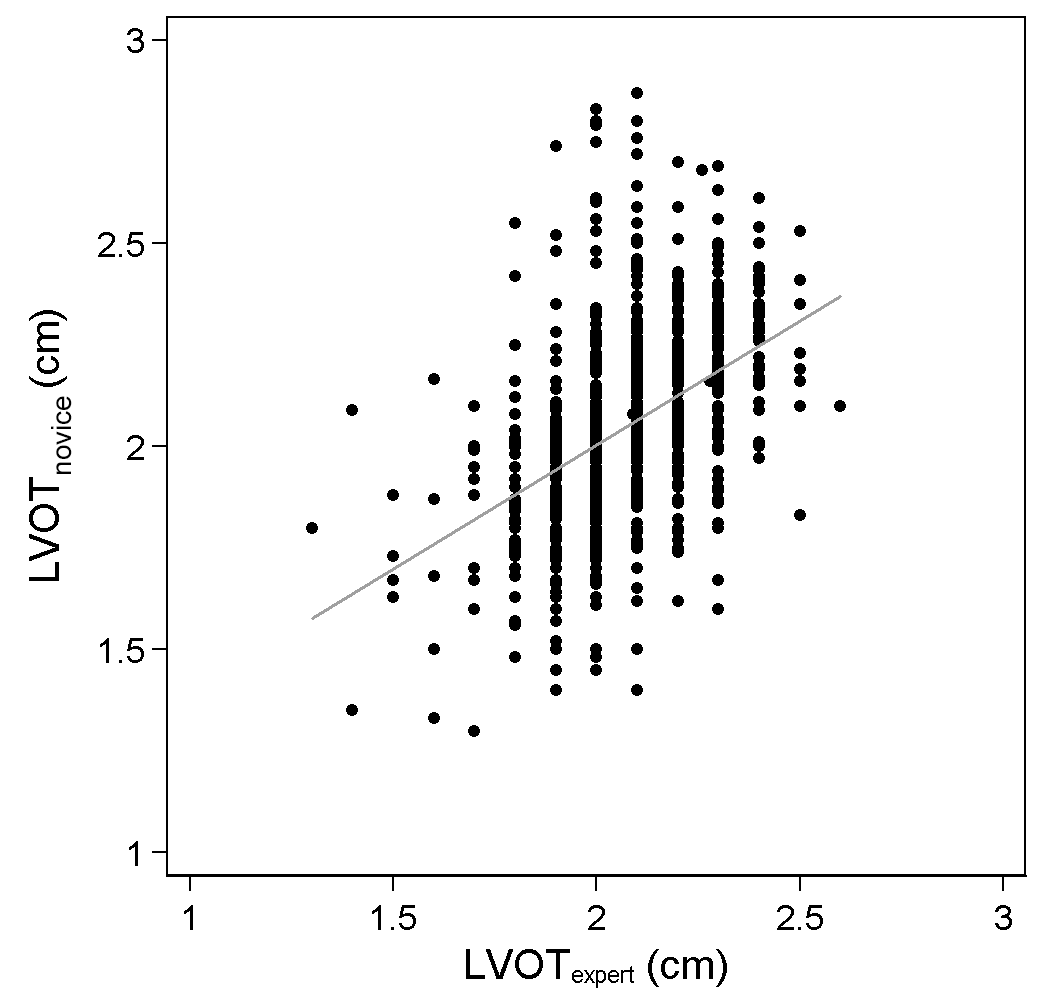


**Figure S4. Bland-Altman plot showing the comparison between LVOT diameter measured by medical students (LVOT_medical students_) and core lab experts (LVOT_expert_).**

**A.**

**
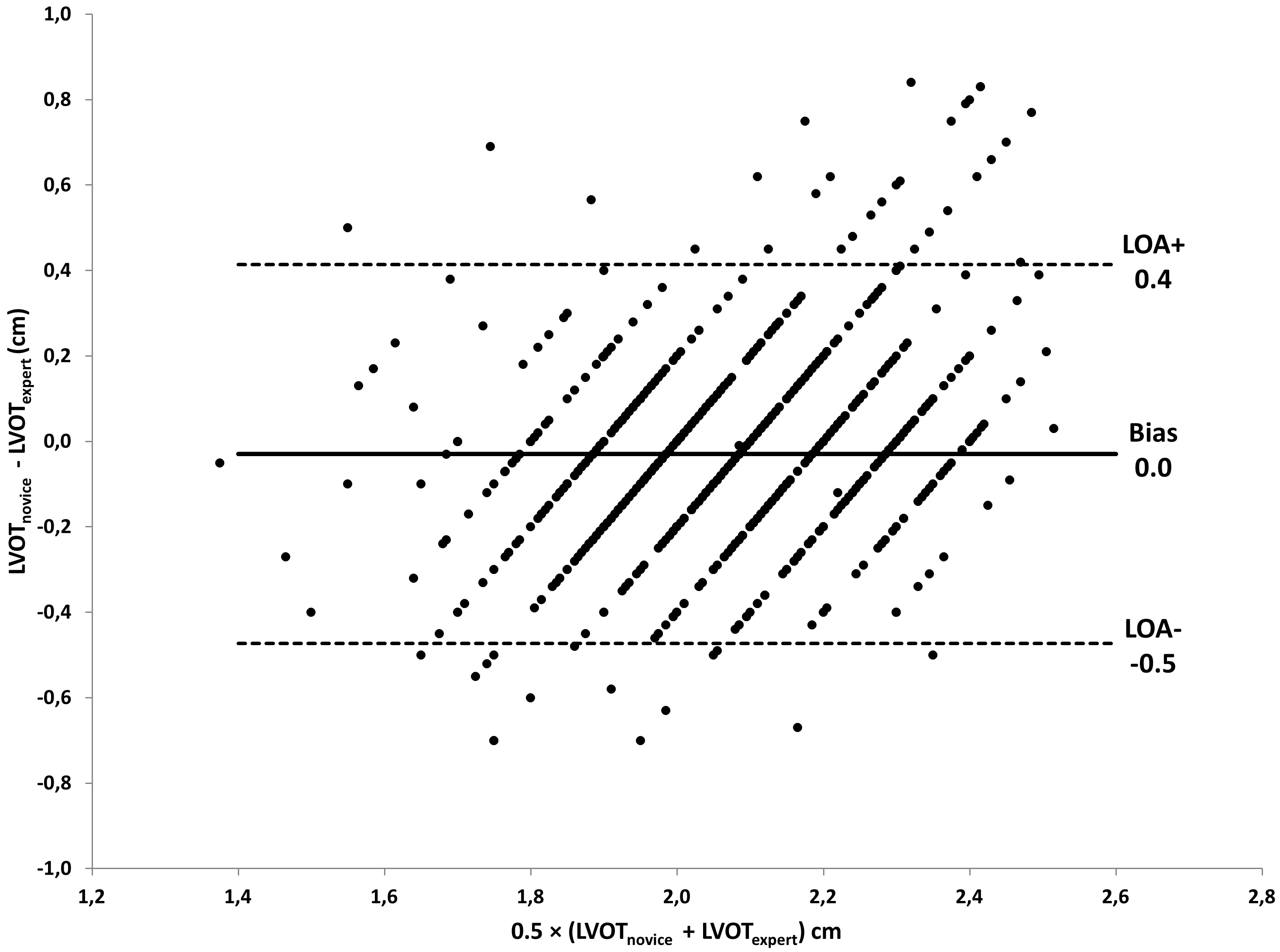
**

**B.**


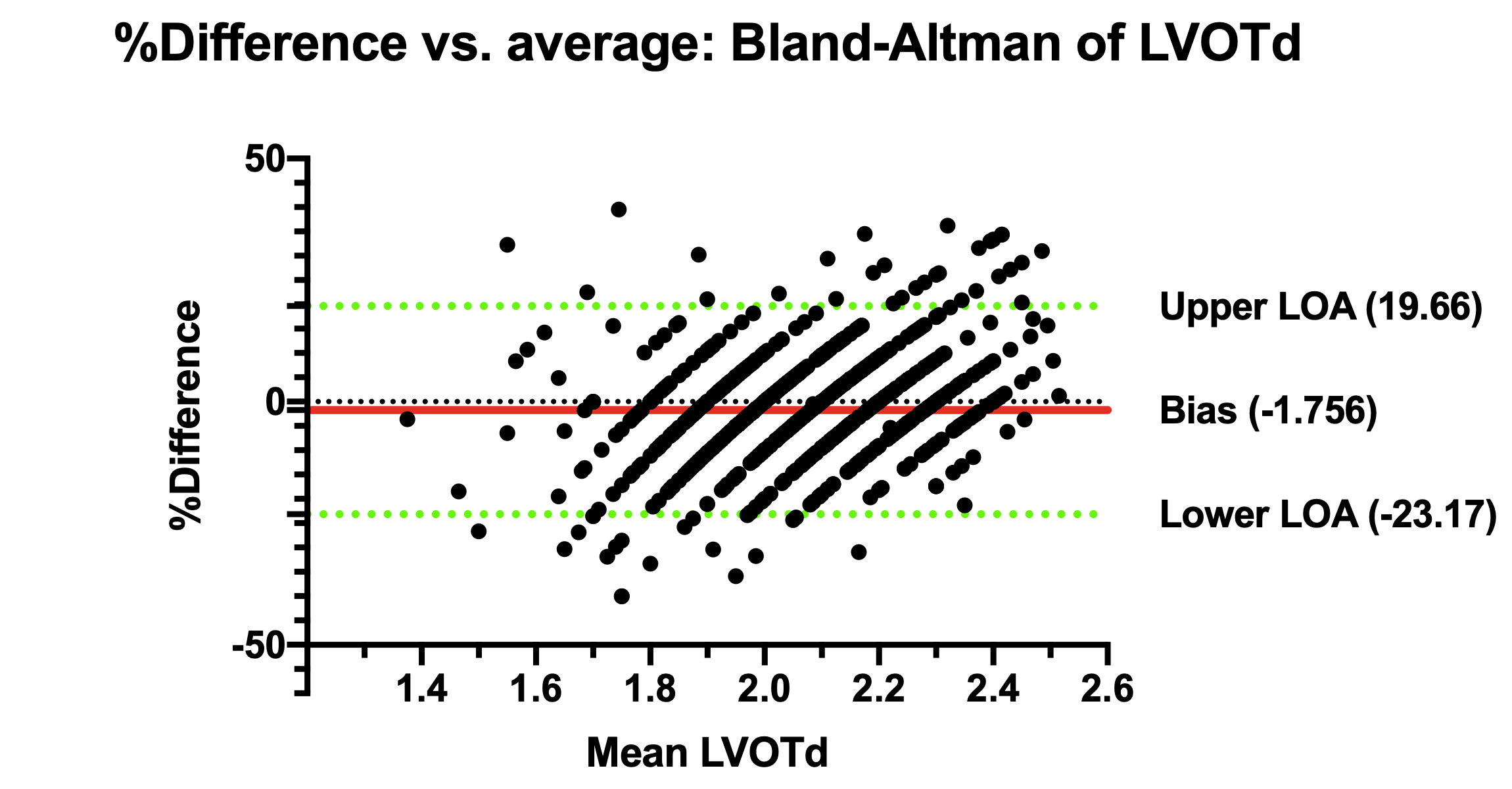


**Legenda**. Bland-Altman plot of absolute difference (A) versus percentage difference (B); the mean bias between LVOT_expert_ and LVOT_medical students_ and the upper and lower limits of agreement (LOA) are presented

**Figure S5. Scatter plot of velocity time interval measurements of medical students (VTI_medical students_) versus core lab (VTI_expert_)**

**
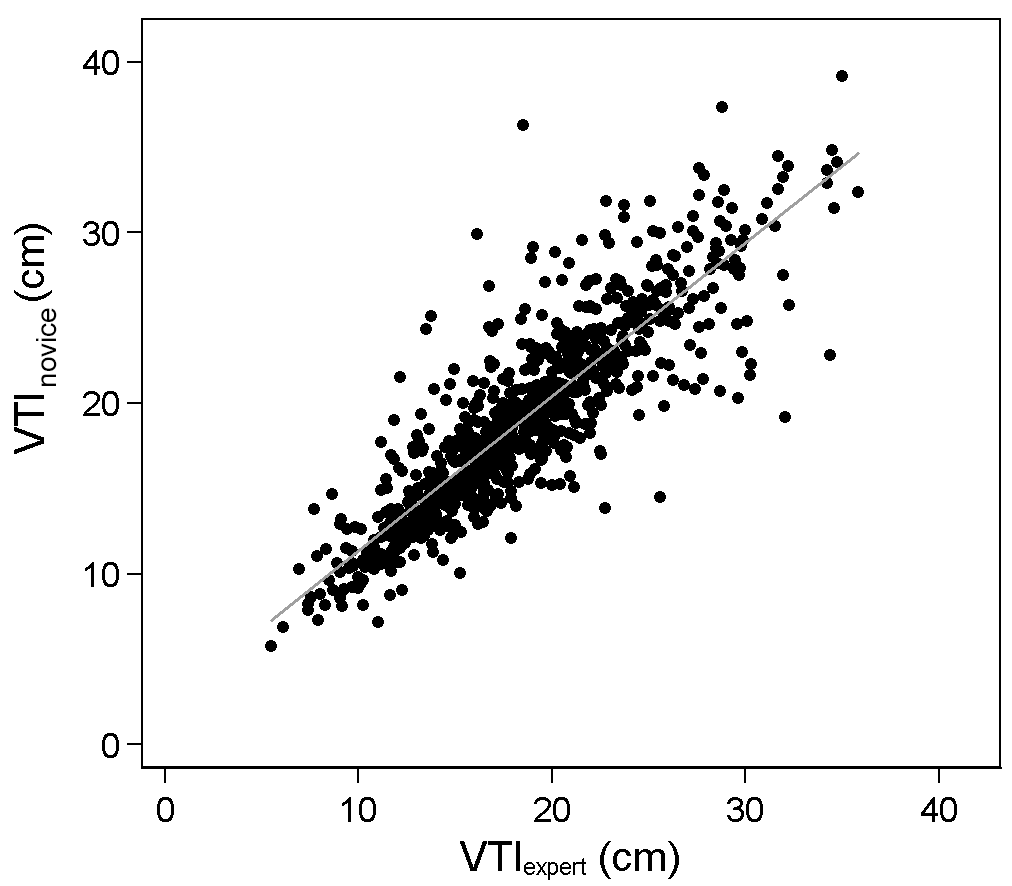
**

**Figure S6. Bland-Altman plot showing the comparison between VTI measured by medical students (VTI_medical students_) and core lab experts (VTI_expert_).**

**A.**

**
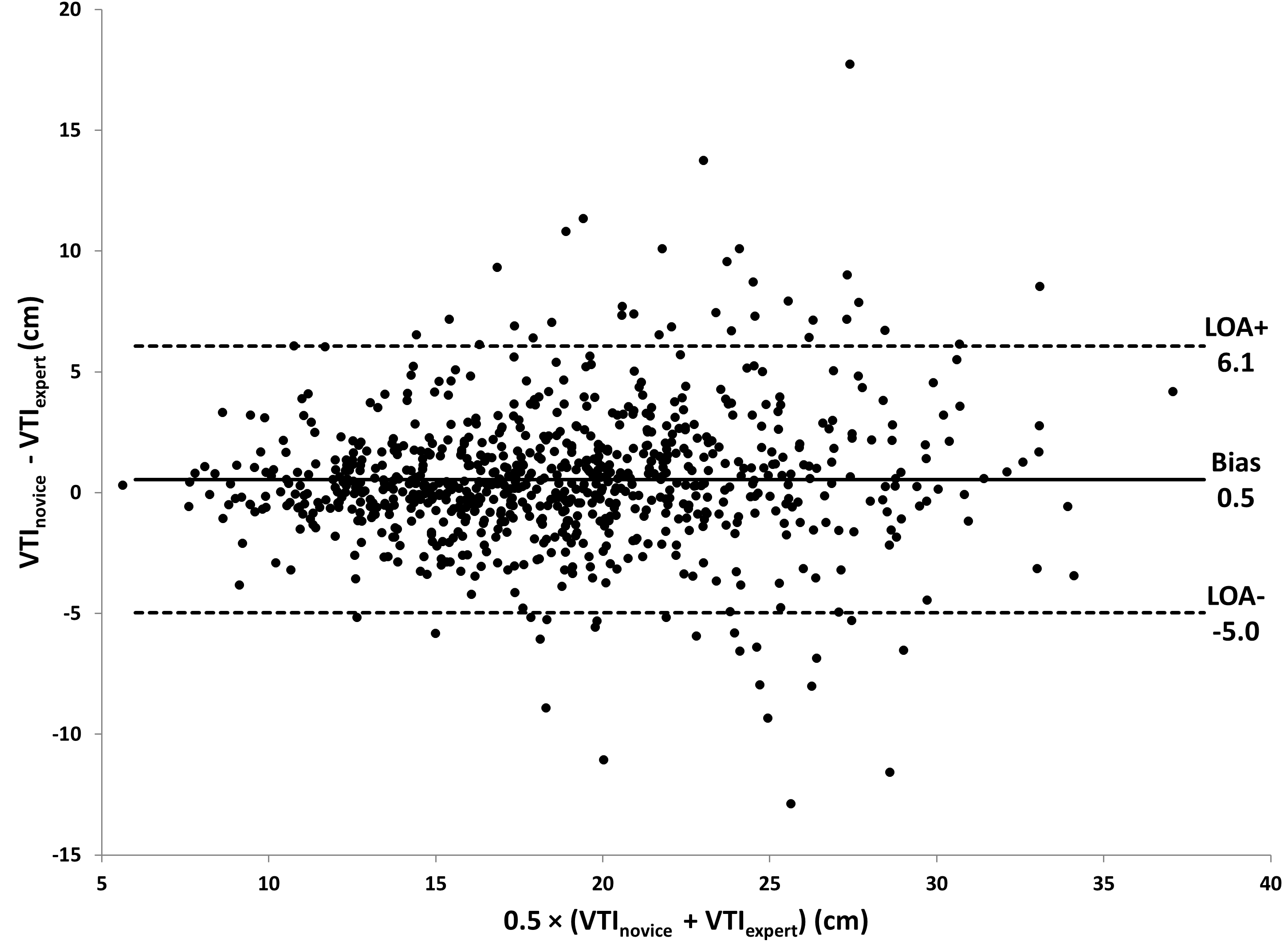
**

**B.**


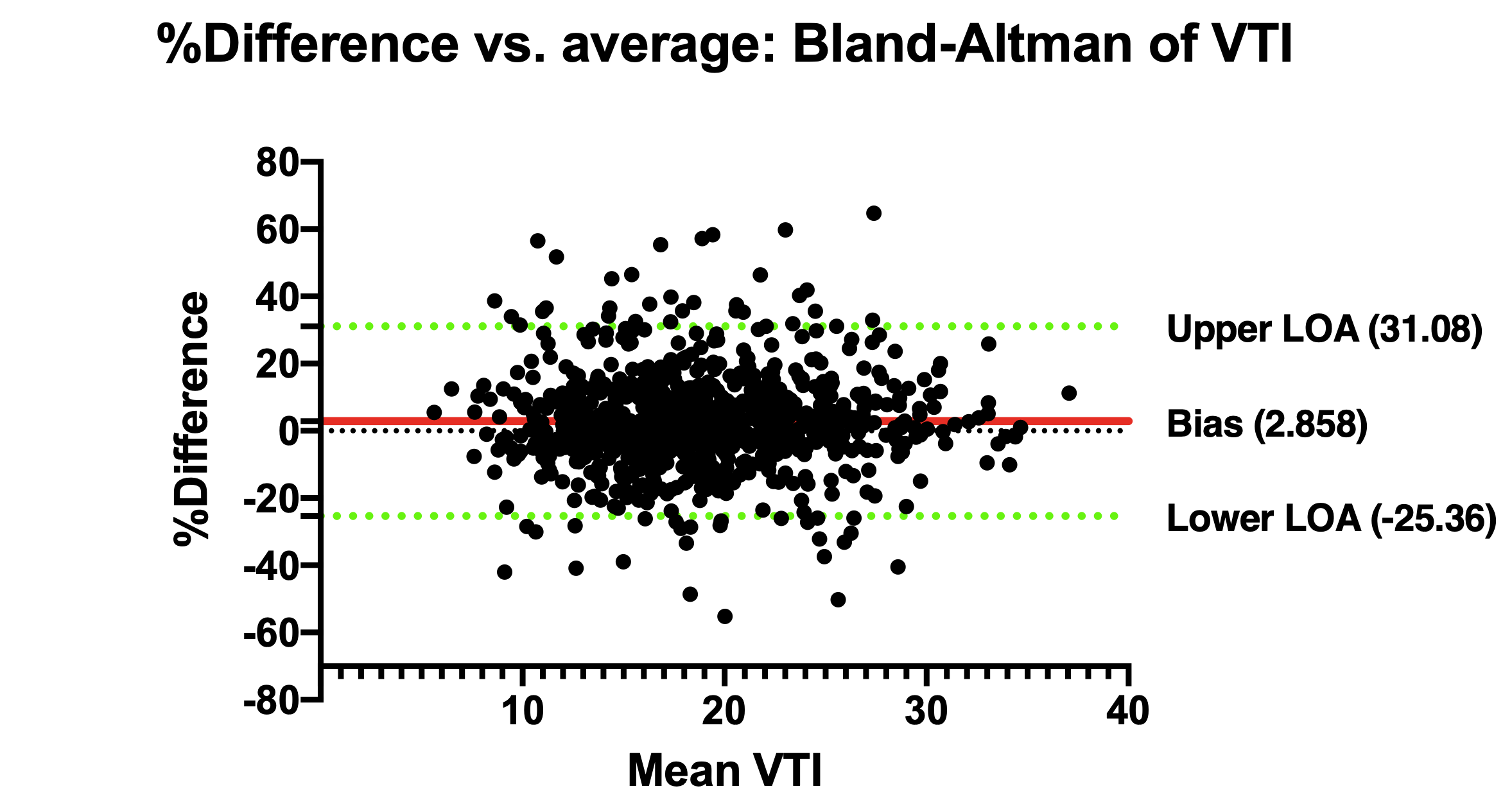


**Legenda**. Bland-Altman plot of absolute difference (A) versus percentage difference (B); the mean bias between VTI_medical students_ and VTI_expert_ and the upper and lower limits of agreement (LOA) are presented

**Table S1. Overview of existing recent literature on medical student-based ultrasonography in critically ill patients**

| **Author, year** | **CTR** | **Setting** | **No of S** | **Operator** | **Patients** | **Eligible** | **No of US** | **CTS** | **MV** | **Population** | **Protocol** | **Image acquisition** | **Image quality** | **Independent judging of images** |
| --- | --- | --- | --- | --- | --- | --- | --- | --- | --- | --- | --- | --- | --- | --- |
| Krause, 2017 | 1 | ED | 148 | Medical students 3^rd^ year surgery clerkship | 444 | ns | 444 | - | - | Pts presented to ED | eFAST exam | ns | ns | No, by an ED resident or attending; agreed with interpretation of student in 99% of the time |
| Udrea, 2017 | 1 | ED | 5 | Medical students end year 1 ^ | 235 of 482 | ns | 641 | - | - | Adult pts in ED ^^ | Cardiac, IVC | 98% (12 of the 641 exams ‘not visualized’) | ns | No, by ED resident; 92% agreement with cardiac findings; 97% agreement with IVC findings |
| *^ Note. Of* a 4-year curriculum with integrated US training; ^^ *Pts who required POCUS (indicated scans) or would consent to receive a scan for educational purposes (training scans)* | | | | | | | | | | | | | | |
| Ho, 2015 | 1 | Anaes-thesia | 133 | Final-year medical students | 246 | ns | 266 | 89% | - | Pts scheduled for surgery, mainly cardiac | Basic TTE by handheld | Students 82% (range 70-92%) ^ | ns | No, by supervisors (anaesthetist or intensivist) |
| *^ Note. Students, when performing TTE, were supervised and provided guidance by the supervisors;* *Supervisors image acquisition was 93% (range 82-98%)* | | | | | | | | | | | | | | |
| Filipiak-Strzecka, 2013 | 1 | ICU | 2 | 4^th^ and 5^th^ year medical student | 90 | ns | 90 | ns | ns | Pts admitted to the cardiac intensive care ^ | Bedside TTE by handheld | 98% (in 1 pt no image could be obtained) | 33% good and 65% acceptable ^^ | No, by cardiologist |
| *^ Note. Pts in unstable condition were excluded from the research if their participation in this study could delay the standard treatment procedures. ^^ Note. In separate group an experienced echocardiographer had good in 63% and acceptable in 37%* | | | | | | | | | | | | | | |
| Panloulas, 2012 | 1 | ED | 8 | 5 final-year medical students/ 3 junior doctors | 122 | ns | 122 (64 by students; 58 by juniors) | - | - | Pts from the cardiology ward and the ED | TTE by pocket-size, handheld | 98% (in 2 pts no image could be obtained) | 89% fair quality; 7.8% poor quality (students/ junior doctors: 91/88%) | No, by TTE accredited cardiologist; The Cohen’s kappa coefficient for LV systolic function was 0.606 (P< 0.001) ^ |
| *^ Note. Sensitivity for moderate-to-severe LV impairment was 74%; specificity 94%* | | | | | | | | | | | | | | |

Abbreviations. CCE = critical care echography; CTR = center; CTS = cardiothoracic surgery; ED = emergency department; eFAST = extended focused assessment with sonography in trauma; ICU = intensive care unit; IVC = inferior vena cava; LV = left ventricle; MV = mechanical ventilation; No = number; ns = not specified; POCUS = point-of-care ultrasound; pts = patients; S = sonographer; TTE = transthoracic echocardiography; US = ultrasound(s)

**Table S2. Overview of existing recent literature on CO derived ultrasonography in critically ill patients compared to our study**

| **Author, year** | **CTR** | **Set-ting** | **No of S** | **Opera-tor** | **US training** | **Patients** | **Eligible** | **No of US** | **Population** | **CTS** | **MV** | **BMI** | **Image acquisi-tion** | **Image obtained** | **Measurement performed** | **Image**  **quality#** | **Averages** | **Pearson correlations** | **Bias, LOA, Percentage error CO** | **Independent judging of images** |
| --- | --- | --- | --- | --- | --- | --- | --- | --- | --- | --- | --- | --- | --- | --- | --- | --- | --- | --- | --- | --- |
| Dingh, 2012 | 1 | ED | 2 | ED physic-cians | 20h | 100 | ns | 100 | Convenience sample of pts presented to ED^ | - | - | ns | ns | 100% for LVOT; 97% for VTI | ns | 3% LVOT unobtainable; 6,2% VTI unobtainable; good LVOT and VTI 90% resp. 78% | CI 2.42Lmin^-1^m^-2^; LVOTd 2.07 cm | CI 0.82, LVOTd 0.80 and VTI 0.87 | -0.11, -1.06 to 0.83, >36 | Yes, by independent cardiologist |
| ^ ED patients that had valvular disease, were unable to lie supine, or were unable to lie in the left lateral decubitus position were excluded | | | | | | | | | | | | | | | | | | | | |
| Lee, 2014 (abstract) | 1 | ED | 2 | Ultra-sound + cardio-logy fellow | ns | 80 | ns | ns | Convenience sample of pts presented to ED | - | - | ns | ns | ns | ns | ns | ns | CO 0.82, LVOT 0.76, VTI 0.66 | 52.5% of pts the 2 operators where within 15% of the  average | ns |
| Betcher, 2018 | 1 | ED | 17 | Junior resident (8); senior resident (7); fellow (2)* | 20min | 53 | ns | 53 | Convenience sample of pts presented  to ED with a chest pain or  dyspnea | - | - | 31 | ns | 68%- 88% for LVOT; 63-88% for VTI | LVOTd 42-82%; VTI 37-65%; both 37-65% | 35-63% LVOTd and VTI unobtainable; adequate LVOT and VTI in 21-59% | - | - | - | Yes, by emergency medicine  ultrasound faculty |
| * All had previous ultrasonography experience | | | | | | | | | | | | | | | | | | | | |
| Villavicencio, 2019 | 1 | ICU | 3 | Intensivists (3) | 20h theory and 10h hands-on | 42 | ns | 42 | Convenience sample of pts admitted to the ICU who required hemodynamic monitoring^ | ns | 90% | 27 | ns | ns | 48% | 33.3% inadequate LVOT and/or VTI | CO 5.22 L/  min (± 1.17 L/min), mean  LVOTd 1.92 cm (± 0.13 cm) and mean VTI 20.85 cm (± 3.72 cm) | CO 0.78 | 1.03, −1.50 to  3.56, 17* | Yes, by independent blinded investigattors |
| ^ Patients were excluded due to atrial fibrillation, aortic valve disease, or technical difficulties in obtaining the PAC-CO measurement; * Ultrasonography derived CO was compared with transpulmonary dilution derived CO | | | | | | | | | | | | | | | | | | | | |
| Koster, 2019 | 1 | ICU | 16 | Medical students year 3-6 | 4h | 1155 | 1442 | 1075 | Consecutive cohort of acutely admitted ICU patients with expected stay>24h | 8% | 59% | 27 | 93% | 81% for LVOT; 82% for VTI | 78% LVOTd; 79% VTI | 21% LVOT unobtainable; 20% VTI unobtainable; good LVOT 80% | CO 5.2 L min^-1^; LVOTd 2.07 cm | CO 0.75, LVOTd 0.45, VTI 0.87 | 0.04, -2.6 to 2.7, 50 | Yes, by echocorelab |

# deemed by independent expert

Abbreviations. CI = cardiac index; CO = cardiac output; CTR = center; CTS = cardiothoracic surgery; ED = emergency department; ICU = intensive care unit; LOA = limits of agreement; LVOT = left ventricular outflow tract; MV = mechanical ventilation; No = number; ns = not specified; pts = patients; S = sonographer; US = ultrasound(s); VTI = velocity time integral

**Appendix: CCUS protocol**

*General outline*

Cardiac output and cardiac index will be measured using transthoracic echocardiography. For study purposes different researchers will be trained in the basics of transthoracic echocardiography by a cardiologist. They will learn how to determine cardiac output by obtaining four different echocardiographic views and subsequent measurements.

*Procedure*

Transthoracic echocardiography will be performed at the bedside during the physical examination with a mobile ultrasonic machine e.g. General Electric Vivid-S6 with the use of the cardiac probe M3S of M4S with default cardiac imaging setting. The patient will be supine or in left lateral tilt (partly on the left). Blood pressure, use of vasopressors and/or inotropic agents, heart rate and rhythm, height and weight will be recorded/noted before examination. After the images have been acquired, cardiac output and cardiac index will be calculated, and the data will be saved on an encrypted hard disk. At a later time, the images will be validated by an echocardiography technician or a cardiologist who will be blinded for all other measurements.

*Views and images*

Three or four standardized echographic views will be obtained in all patients:

1. Parasternal long axis view (PLAX);
2. parasternal short axis view (PSAX);
3. apical four chamber view (AP4CH);
4. apical five chamber view (AP5CH).

The PSAX view will only be obtained in case the PLAX does not provide a clear image of the aortic annulus. The views are described in more detail below.


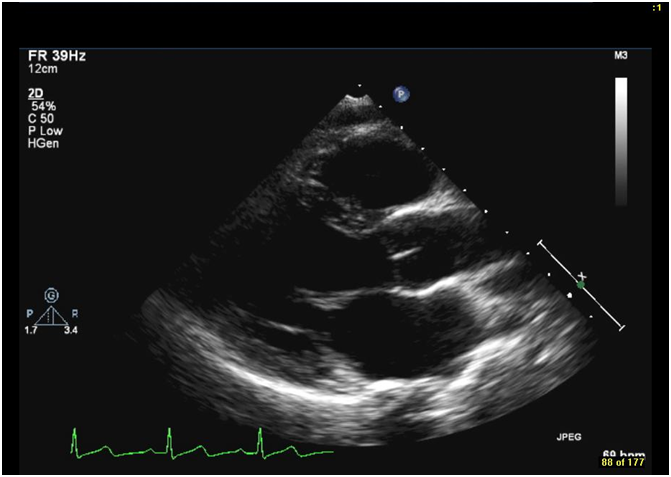


Figure 2. Parasternal long axis (PLAX)

*Parasternal long axis (PLAX)*

The parasternal window is located next to the sternum, between the 3rd and 5th intercostal space.

Criteria of quality for a good view (figure 2):

- Minimized angle between ascending aorta and left ventricle;
- maximized width view of left ventricle;
- maximal opening of mitral valve (showing both anterior and posterior mitral valve leaflets, right- and noncoronary cusps of aortic valve;
- no papillary muscle in view.

The PLAX view is the primary view used to measure the left ventricular outflow tract (LVOT). An image will be saved for validation.

Figure 3. Parasternal short axis (PSAX)


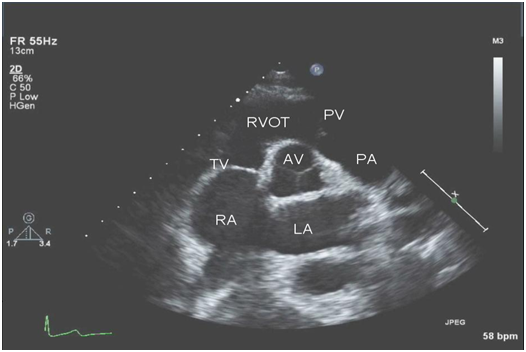


*Parasternal short axis (PSAX)*

This view will only be obtained in case the PLAX view does not provide a clear image of the LVOT. The PSAX view can be obtained on several levels. For study purposes it will be measured on the aortic, tricuspid and pulmonic valve level (figure 3). An image will be saved for validation.

*Apical four and five chamber view (AP4CH and AP5CH)*

Figure 4. Apical four chamber view (AP4CH)


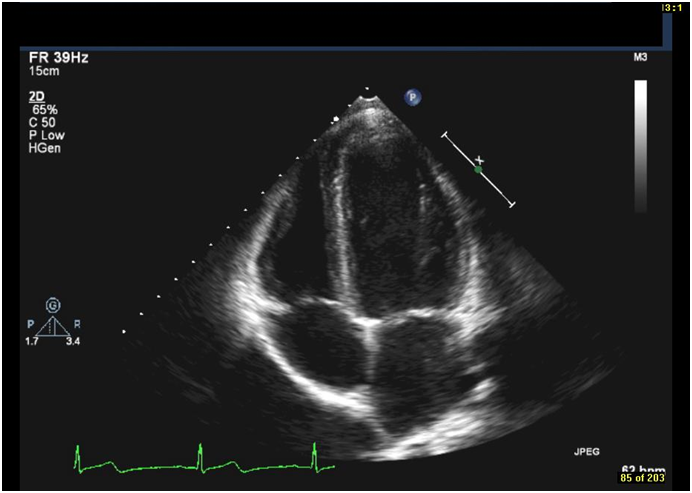


The apical echographic window is located at the apex of the left ventricle (apical impulse).

Criteria of quality for a good view (figure 4, 5):

- Maximized view of endocardial border;
- FPS > 40;
- the entire endocardium is within scan sector in *both end-diastole and end-systole*;


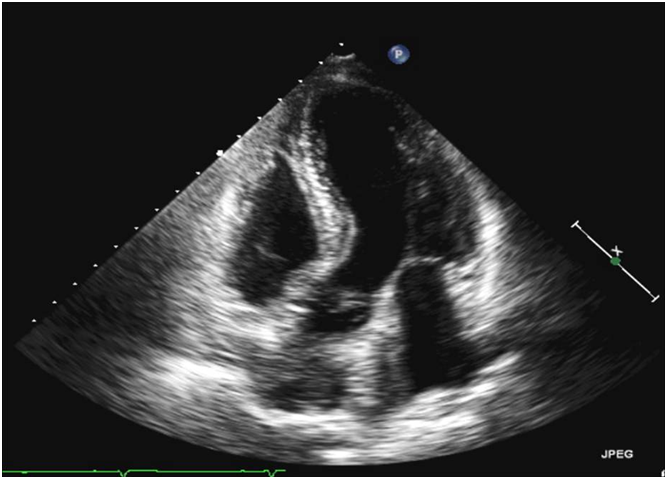


**Figure 5.** Apical five chamber view (AP5CH)

- avoid apical foreshortening.

From the four chamber view the probe will be tilted caudally to obtain the apical five chamber view. In the apical five chamber view the velocity time integral will be measured using the pulse wave Doppler signal from the LVOT. Of both views an image will be saved for validation.

*Measuring the left ventricular outflow tract (LVOT) and the velocity time integral (VTI)*

The LVOT diameter changes very little through systole and diastole and is assumed to be constant and closely approximating a circle in shape. The LVOT diameter will be measured in 2D in the parasternal long axis view in systole (figure 7). If a clear image cannot be obtained through this view, the LVOT will be measured in the parasternal short axis view.

The LVOT velocity time integral (LVOT-VTI) provides information regarding blood flow velocity across the time period of systole and is in the units of cm. Typical values are close to 2 cm. Blood flow velocity will be measured just above the aortic valve in the apical five chamber view by using pulse wave Doppler. The velocity time integral will be traced out on the ultrasound machine (figure 7). In case of an irregular rhythm such as atrial fibrillation, the average VTI of several beats will be used. Images of both measures will be saved for validation.


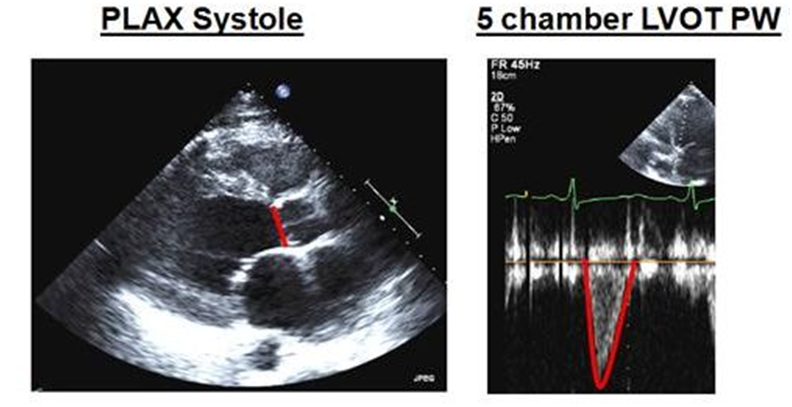


Figure 7. *Left:* the left ventricular outflow tract (LVOT). *Right:* the velocity time integral (VTI).

*Calculating cardiac output and cardiac index*

Cardiac output will be automatically calculated on the ultrasound machine after measuring the LVOT, VTI and heart rate. Cardiac index will be automatically derived using patient length and weight.

*Data management*

The echocardiographic images will be saved to an encrypted USB drive. This USB drive will solely be used for research purposes. After the examination the USB drive is used to transfer images to a UMCG computer where they will be uploaded to the data management system (OpenClinica) and stored on the central secure server. After the upload has been confirmed, files on the USB drive will be deleted. The following four images will be uploaded to OpenClinica:

| **View** | **What will be saved?** |
| --- | --- |
| PLAX | Image |
| PLAX | LVOT diameter measurement (figure 7) |
| AP5CH | Image |
| AP5CH | Velocity time integral measurement (figure 7) |

A more complete image series will be saved to the internal hard disk of the echo Doppler machine. This is required for later validation.
